# Quantitative second harmonic generation microscopy for characterizing collagen remodeling in papillary thyroid carcinoma

**DOI:** 10.1117/1.JBO.31.5.056501

**Published:** 2026-05-25

**Authors:** Wesley Poon, Lin Wang, Orhun Davarci, Reid Master, Hong Zhao, Jun Liu, Helmi S. Khadra, Elizabeth M. Jacobi, Raksha Raghunathan, Stephen T. C. Wong

**Affiliations:** a Houston Methodist Neal Cancer Center, Department of Systems Medicine and Bioengineering, Houston, Texas, United States; b Texas A&M University, College of Medicine, Texas A&M Health Science Center, Bryan, Texas, United States; c Houston Methodist Neal Cancer Center, T. T. & W. F. Chao Center for BRAIN, Department of Systems Medicine and Bioengineering, Houston, Texas, United States; d Texas A&M University, School of Engineering Medicine, Houston, Texas, United States; e Houston Methodist Research Institute and Houston Methodist Neal Cancer Center, Advanced Cellular and Tissue Microscopy Shared Resource, Houston, Texas, United States; f Shanghai General Hospital, Shanghai Jiao Tong University, Department of Breast-Thyroid-Vascular Surgery, Shanghai, China; g Houston Methodist Hospital, Department of Surgery, Houston, Texas, United States; h Houston Methodist Hospital, Department of Pathology and Genomic Medicine, Houston, Texas, United States; i Houston Methodist Hospital, Department of Radiology, Houston, Texas, United States

**Keywords:** second harmonic generation microscopy, thyroid cancer, label-free imaging, nonlinear optical imaging

## Abstract

**Significance:**

Thyroid cancer is the most common endocrine malignancy. Although fine needle aspiration (FNA) is the gold standard for diagnosis, 10% to 20% of FNAs yield indeterminate cytology, of which 20% to 30% prove malignant. Such diagnostic uncertainty drives repeat FNAs, costly molecular tests, and unnecessary surgeries, leading to increased healthcare costs, reduced quality of life, and emotional distress to patients. Current methods provide limited structural information about the extra-cellular matrix, despite collagen remodeling being a hallmark of malignancy. A rapid, label-free, and architecture-based diagnostic approach has the potential to significantly improve real-time or near-real-time decision-making and reduce unnecessary biopsies and interventions.

**Aim:**

We use a systematic, quantitative framework based on second harmonic generation (SHG) microscopy to differentiate between normal and cancerous (papillary thyroid carcinoma [PTC]) human thyroid tissues through collagen architecture signatures.

**Approach:**

Formalin-fixed paraffin-embedded unstained tissue sections (5  μm thick) of confirmed PTC and normal thyroid tissue were obtained under an approved IRB and imaged under a multiphoton microscope using an excitation wavelength of 803 nm. An emission filter of 400±10  nm was used to filter the SHG emission from the sample. Thirty-five metrics capturing intensity, geometric, textural, and frequency-domain collagen features were quantified. A linear mixed-effects model was used for quantitative comparison between cancer and normal groups, accounting for the hierarchical structure of the data and rigorously assessing metric significance despite limited sample size.

**Results:**

Of the 35 metrics evaluated, 19 remained statistically significant after false discovery rate (FDR) adjustment for multiple comparisons. The corresponding effect sizes ranged from moderate to large, indicating substantial separation between normal and cancerous samples. Five of the remaining 16 metrics, although not statistically significant, were consistently either higher or lower in one group compared with the other, suggesting directional consistency. This revealed robust and biologically coherent alterations in collagen organization despite small cohort size. These included consistent increases in spectral power across multiple spatial frequency bands and greater collagen heterogeneity in cancer tissue.

**Conclusions:**

The convergence of significant and directionally consistent metrics underscores the strong diagnostic potential of SHG-derived collagen architecture. Integrating these quantitative features into an automated analysis platform could enable rapid, objective, and reproducible optical biopsies, with the potential to reduce indeterminate FNAs, guide intraoperative decision-making, and improve diagnostic accuracy in routine thyroid cancer care.

## 1 Introduction

Thyroid cancer ranks as the ninth most common cancer worldwide and accounts for 95% of all endocrine tumors.^1^^,^^2^ It can develop at any age but is the leading malignancy among adolescents and young adults aged 16 to 33 years. Based on histopathology, thyroid cancers are classified into three main categories^3^: (a) differentiated thyroid cancers, which include papillary, follicular, and oncocytic subtypes; (b) medullary thyroid cancer; and (c) anaplastic thyroid cancer. Among these, papillary thyroid carcinoma (PTC) is the most prevalent, accounting for ∼80% of all thyroid cancers.^3^^,^^4^

Thyroid cancer is often first detected as a palpable nodule in 30% to 40% of cases.^5^^–^^7^ In recent years, the widespread use of modalities such as ultrasound, computed tomography (CT), and magnetic resonance imaging (MRI) has led to increased detection of incidental thyroid nodules.^5^^,^^8^^,^^9^ However, this trend has contributed to overdiagnosis of low-risk cancers and benign conditions, as well as false positives, resulting in unnecessary surgeries and treatments. These interventions have risks of complications and potential need for thyroid hormone supplementation, which further increase healthcare costs^10^ and reduce patients’ quality of life.^11^^,^^12^ To mitigate overdiagnosis, evidence-based guidelines now advise against routine thyroid ultrasound in asymptomatic individuals,^13^ including those with hyperthyroidism or hypothyroidism but without a palpable thyroid nodule.

The current gold standard for diagnosing thyroid cancer is fine-needle aspiration (FNA), with core needle biopsy (CNB) preferred for rapidly enlarging neck masses.^14^ To reduce overdiagnosis of small, potentially indolent tumors, international risk stratification systems guide the selection of nodules requiring FNA by estimating malignancy risk.^3^ However, some cancers and nodules lack characteristic sonographic features, making biopsy indispensable for accurate diagnosis.^15^^,^^16^ As a result, over 600,000 FNAs are still performed annually in the United States, many of which are unnecessary. FNA also has notable inherent limitations, including indeterminate cytology,^17^^–^^19^ sampling variance, and false negative results. Patients with inconclusive findings often undergo repeat FNA, molecular testing, CNB, or even diagnostic surgery. Although molecular testing can improve diagnostic accuracy, it is costly and often inaccessible in low-resource settings. CNB provides larger tissue samples than FNA but still produces 6.4% to 26.7% inconclusive results and carries risks when performed near critical structures such as the carotid artery.^20^^–^^25^ Furthermore, histopathological analysis of biopsy specimens is labor-intensive, alters native tissue morphology, and is subject to both inter- and intraobserver variability. Together, these limitations highlight the pressing need for a more accurate, rapid diagnostic tool capable of distinguishing normal thyroid tissue, benign conditions and neoplasms, and malignant thyroid tissue, thereby overcoming the shortcomings of conventional cytology, molecular testing, and histopathology.

Multiple studies have demonstrated that the tumor microenvironment (TME) plays a critical role in the initiation, progression, and therapeutic resistance of thyroid cancer.^26^ The TME comprises the extracellular matrix (ECM), soluble signaling factors, metabolites, blood and lymphatic vessels, and diverse stromal cell populations, all of which dynamically interact with tumor cells to drive ECM remodeling, tumorigenesis, and malignant progression.^27^ As a result, TME-based biomarkers have emerged as promising candidates for improving the accuracy and robustness of thyroid cancer diagnosis.

Among TME components, collagen is particularly important. As the predominant structural protein in the ECM, it undergoes marked biochemical, architectural, and organizational alterations during cancer development and progression.^28^ Second harmonic generation (SHG) microscopy, a well-established nonlinear optical imaging technique, enables label-free, high-resolution visualization of collagen architecture in tissue.^29^^–^^31^ Quantitative characterization of collagen alterations using SHG microscopy, such as changes in fiber orientation, density, or anisotropy, may provide a reliable biomarker for distinguishing benign from malignant thyroid tissue,^32^^–^^34^ thereby enhancing diagnostic precision in thyroid cancer.

In this study, we applied SHG microscopy to image both normal and malignant human thyroid tissues and evaluated a comprehensive panel of quantitative collagen metrics. To achieve accurate tissue differentiation, we introduced a suite of 35 metrics categorized into four different groups that, to our knowledge, have not previously been utilized together for thyroid cancer identification. Among these, nineteen metrics demonstrated statistically significant differences between normal and cancerous thyroid tissues. Five out of the remaining 16 metrics exhibited directionally consistent trends, remaining either consistently higher or lower in cancer samples compared with normal samples, although they did not reach statistical significance. Overall, the strong directional consistency observed across the majority of metrics highlights the potential of our quantitative SHG-based analytical methods to distinguish structural alterations associated with thyroid malignancy.

## 2 Materials and Methods

### 2.1 Tissue Samples

Formalin-fixed paraffin-embedded unstained tissue sections (5  μm thick) of confirmed PTC and normal thyroid tissue were obtained from Shanghai General Hospital, Shanghai, People’s Republic of China, following an institutional review board approval. Five samples from each group were selected for imaging. Unstained slides were used for SHG imaging, whereas adjacent consecutive sections were stained with hematoxylin and eosin (H&E) for histopathological validation. The H&E slides were reviewed by a board-certified pathologist, who confirmed the diagnoses of normal thyroid tissue and PTC. Furthermore, all PTC specimens were verified to have been biopsied from the tumor core.

### 2.2 Imaging System and Acquisition

A dual-output ultrafast tunable laser (Insight DeepSee, Santa Clara, USA) was used as the excitation source of the imaging system. The tunable wavelength source was set to 803 nm and linearly polarized for SHG imaging. Samples were imaged using a Bergamo multiphoton microscope (Thorlabs, Inc., NJ, USA) at 25× magnification. An emission filter of 400±10  nm was used to filter the light from the sample, and a photomultiplier tube was used to collect the light. SHG signals were acquired both in the forward and backward directions for all samples. However, aligning with the translational direction of this work, all quantifications were made on the backward detected images. Forward-detected images were used only for forward-to-backward ratio-based calculations (detailed in Sec. 2.5).

For each tissue specimen, five random fields of view (FOVs) were selected for imaging, provided that the majority of the region contained collagen signals. Each FOV consisted of a 512×512  pixel grid, with a pixel size of 0.94  μm, corresponding to an imaging area of ∼430  μm×430  μm per FOV. At each FOV, images were acquired along the entire depth of the slide and at each depth, four frames were acquired and averaged to improve the signal-to-noise ratio (SNR). Finally, a maximum intensity projection (MIP) was subsequently generated across the depth stack for each FOV along depth, and the resulting MIP image was used for quantitative analysis. As a preprocessing control, a region of the glass slide without tissue was imaged after completing data acquisition for each specimen.

### 2.3 Preprocessing of SHG Images

To suppress salt-and-pepper noise, a 3×3 two-dimensional median filter was applied to each MIP image. For each sample, the 99th percentile signal from the corresponding glass-only FOV was then subtracted from the median-filtered sample FOV, with negative intensity values set to zero. This approach provided a robust measure of noise without substantially overestimating it due to occasional PMT spikes while also accounting for most of the salt-and-pepper noise. The resulting background-corrected images were subsequently normalized to a 0 to 1 scale. FOVs in which the majority of pixels represented slide background, operationally defined as fewer than 5% of pixels retaining a nonzero signal after background noise reduction, were excluded from downstream quantitative analysis.

### 2.4 Data Analysis and Statistical Testing

The dataset comprised samples from five normal and five cancer patients, with five random FOVs imaged per sample, yielding 25 FOVs per group. Quantitative metrics were computed for each FOV as detailed in Sec. 2.5. To account for the hierarchical structure of the data (multiple FOVs per patient), we employed a linear mixed-effects model (LMM) with a random intercept model structure for all quantitative comparisons between cancer and normal tissue. Group (cancer versus normal) was specified as a fixed effect, and patient ID was included as a random intercept to account for intrapatient correlation. To quantify the magnitude of group differences, standardized effect sizes were estimated using model-based standardized coefficients derived via the refit method in the effectsize package.^35^ This approach provides a standardized mean difference analogous to Cohen’s d, adjusted for the variance components of the mixed-effects model.^36^ To assess the stability of each metric across FOVs within the same patient, we calculated the adjusted intraclass correlation coefficient (ICC), defined as:

$$\mathrm{ICC}=\frac{\sigma_{\text{patient}}^{2}}{\sigma_{\text{patient}}^{2}+\sigma_{\text{residual}}^{2}},$$ (1)

where variance components were extracted from the fitted mixed model after accounting for the group effect.

Statistical significance of fixed effects was evaluated using Satterthwaite’s approximation of degrees of freedom. To account for multiple comparisons across the 35 quantitative metrics, P values were adjusted using the Benjamini–Hochberg false discovery rate (FDR) procedure, and FDR-adjusted P<0.05 was considered statistically significant. All analyses were conducted in R (version 4.5.1) using the lme4, lmerTest, and effectsize packages.

### 2.5 Quantitative Metrics for Feature Extraction

#### 2.5.1 Collagen area fraction

Collagen area fraction (CAF) is defined as the ratio of collagenous area compared with the total area of the FOV. To calculate CAF, a binary map was created where all pixel values above intensity 0 are mapped to logical 1, and every other pixel is mapped to logical 0. The total number of pixels mapped to logical 1 divided by the total number of pixels within a FOV is considered CAF.

#### 2.5.2 Forward to backward ratio

SHG directionality was quantified using the forward-to-backward (F/B) ratio. For each FOV, the forward-channel image was divided by the corresponding backward-channel image through pixel-wise matrix division, where each pixel intensity in the forward image was divided by the corresponding pixel intensity in the backward image. Pixel-wise F/B ratio values were averaged to obtain a representative F/B ratio for each FOV. To prevent division-by-zero errors, a small epsilon value of 0.001 was introduced. Following normalization, pixels with forward or backward intensity values below 0.001 were discarded and excluded from the FOV-level averaging. In addition, the ratio of CAF (CAF F/B) for the forward and backward channels was also computed to compare directional differences in collagen organization.

#### 2.5.3 Gray-level co-occurrence matrix

The gray-level co-occurrence matrix (GLCM) captures spatial relationships among pixels for texture analysis.^37^^–^^39^ In this study, GLCM-based metrics were employed for feature extraction, building on approaches previously used to distinguish cancerous from normal tissues.^40^^,^^41^ To preserve the high resolution of SHG microscopy and the fine structure of collagen fibers, images were represented using 64 gray levels. To create the GLCMs, three distances (d=1,2,3) were used along each of the axes $\theta_{p}=[0,45,90,135]$. Using the GLCMs generated for each image, a total of 21 texture features were extracted, as summarized in Table 1. The *graycomatrix* function from the Image Processing Toolbox in MATLAB R2024a (MathWorks, Natick, MA, USA) was used for this analysis. Contrast, dissimilarity, homogeneity, energy, entropy, and maximum probability describe intensity contrast, smoothness, and uniformity based on pixel co-occurrences. Correlation and autocorrelation quantify gray-level linear dependencies. Cluster prominence, cluster shade, and cluster tendency represent joint statistical moments of intensity pairs. Features derived from the sum and difference matrices were evaluated using their mean, variance, and entropy. Information measures of correlation (IMC) quantify the mutual dependence between row and column marginal distributions, whereas inverse difference metrics characterize local homogeneity by reducing the influence of gray-level variations.

**Table 1:** Explored GLCM features.

GLCM features	Equation
Autocorrelation	$\overset{N_{g}}{\underset{i=1}{\sum }}\overset{N_{g}}{\underset{j=1}{\sum }}p(i,j)ij$
Contrast	$\overset{N_{g}}{\underset{i=1}{\sum }}\overset{N_{g}}{\underset{j=1}{\sum }}(i-j{)}^{2}p(i,j)$
Correlation	$\frac{\sum_{i=1}^{N_{g}}\sum_{j=1}^{N_{g}}p(i,j)ij-\mu_{x}\mu_{y}}{\sigma_{x}(i)\sigma_{y}(j)}$
Cluster prominence	$\overset{N_{g}}{\underset{i=1}{\sum }}\overset{N_{g}}{\underset{j=1}{\sum }}(i+j-\mu_{x}-\mu_{y}{)}^{4}p(i,j)$
Cluster shade	$\overset{N_{g}}{\underset{i=1}{\sum }}\overset{N_{g}}{\underset{j=1}{\sum }}(i+j-\mu_{x}-\mu_{y}{)}^{3}p(i,j)$
Cluster tendency (i.e., sum variance)	$\sum_{i=1}^{N_{g}}\sum_{j=1}^{N_{g}}(i+j-\mu_{x}-\mu_{y}{)}^{2}p(i,j)$
Dissimilarity (i.e., Difference average)	$\sum_{i=1}^{N_{g}}\sum_{j=1}^{N_{g}}|i-j|p(i,j)$
Joint energy (i.e., angular second moment)	$\sum_{i=1}^{N_{g}}\sum_{j=1}^{N_{g}}(p(i,j){)}^{2}$
Entropy	$-\overset{N_{g}}{\underset{i=1}{\sum }}\overset{N_{g}}{\underset{j=1}{\sum }}p(i,j)\log_{2}(p(i,j)+\epsilon )$
Homogeneity (i.e., inverse difference)	$\overset{N_{g}}{\underset{i=1}{\sum }}\overset{N_{g}}{\underset{j=1}{\sum }}\frac{p(i,j)}{1+|i-j|}$
Maximum probability	max(p(i,j))
Sum of squares (variance)	$\sum_{i=1}^{N_{g}}\sum_{j=1}^{N_{g}}(i-\mu_{x}{)}^{2}p(i,j)$
Sum average	$\overset{2N_{g}}{\underset{k=2}{\sum }}kp_{x+y}(k),\;k=x+y$
Sum entropy	$-\overset{2N_{g}}{\underset{k=2}{\sum }}p_{x+y}(k)\log_{2}(p_{x+y}(k)+\epsilon ),\;k=x+y$
Difference variance	$\overset{N_{g}-1}{\underset{k=0}{\sum }}(k-\mathrm{DA}{)}^{2}p_{x-y}(k)$, where DA = Difference average (i.e., Dissimilarity)
Difference entropy	$\overset{N_{g}-1}{\underset{k=0}{\sum }}p_{x-y}(k)\log_{2}(p_{x-y}(k)+\epsilon ),\;k=x-y$
Information measure of correlation 1	$\mathrm{HXY}=\overset{N_{g}}{\underset{i=1}{\sum }}\overset{N_{g}}{\underset{j=1}{\sum }}p(i,j)\log_{2}(p(i,j)+\epsilon )$
	$\mathrm{HXY}1=-\overset{N_{g}}{\underset{i=1}{\sum }}\overset{N_{g}}{\underset{j=1}{\sum }}p(i,j)\log_{2}(p_{x}(i)p_{y}(j)+\epsilon )$
	$\mathrm{HX}=-\overset{N_{g}}{\underset{i=1}{\sum }}p_{x}(i)\log_{2}(p_{x}(i)+\epsilon ),\;\mathrm{HY}=-\overset{N_{g}}{\underset{j=1}{\sum }}p_{y}(j)\log_{2}(p_{y}(j)+\epsilon )$
	$\mathrm{IMC}1=\frac{\left(\mathrm{HXY}-\mathrm{HXY}1\right)}{\max(\mathrm{HX},\mathrm{HY})}$
Information measure of correlation 2	$\mathrm{HXY}2=-\overset{N_{g}}{\underset{i=1}{\sum }}\overset{N_{g}}{\underset{j=1}{\sum }}p_{x}(i)p_{y}(j)\log_{2}(p_{x}(i)p_{y}(j)+\epsilon )$
	$\mathrm{IMC}2=\sqrt{1-e^{-2*(HXY2-HXY)}}$
Inverse difference normalized	$\overset{N_{g-1}}{\underset{k=0}{\sum }}\frac{p_{x-y}(k)}{1+(\frac{k}{N_{g}})}$
Inverse difference moment normalized	$\overset{N_{g-1}}{\underset{k=0}{\sum }}\frac{p_{x-y}(k)}{1+(\frac{k^{2}}{N_{g}^{2}})}$
Inverse variance	$\sum_{k=1}^{N_{g}-1}\frac{p_{x-y}(k)}{k^{2}}$

Table 1 also shows the mathematical formulations of all GLCM features and provides interpretations indicating how relatively large values for each independent metric reflect specific texture properties. Each feature is computed from normalized co-occurrence probabilities as follows:

$$P(i,j)=\frac{\mathrm{GLCM}(i,j)}{\underset{i,j}{\sum }\mathrm{GLCM}(i,j)},$$ (2)

where marginal distributions $p_{x}(i)=\underset{j}{\sum }=P(i,j)$ and $p_{y}(j)=\underset{i}{\sum }=P(i,j)$ for which means $\mu_{x}$ and $\mu_{y}$ and standard deviations $\sigma_{x}$ and $\sigma_{y}$ can be derived for the gray level $N_{g}$. Probability of gray-level difference k is defined as

$$p_{\left|i-j\right|}(k)=\underset{|i-j|=k}{\sum }P(i,j),\;k\in \{0,1,\ldots ,N_{g}-1\}.$$ (3)

#### 2.5.4 Orientation-dependent gray level co-occurrence matrix

While GLCM enables quantitative analysis of collagen fibers, it does not account for the geometric arrangement of fiber bundles. Although GLCM metrics are computed across four directions, the dominant orientation of collagen fibers is typically not considered. Given the importance of collagen fiber orientation, incorporating it into GLCM analysis could enhance morphological characterization and provide deeper insights into underlying pathological processes.

Orientation-dependent GLCM (OD-GLCM) aims to generate higher-order features that quantify variations in the linearity of structural patterns.^42^ It primarily evaluates how the GLCM correlation metric changes with respect to angular variations in the orientation of linear structures. Traditional OD-GLCM computes a single correlation value (C) for each direction at a fixed distance. However, this approach may be influenced by artifacts arising from artificially elevated correlation values at larger pixel distances. To mitigate this effect, we calculated the cumulative sum of GLCM correlation values across multiple distances for each angle, treating the discrete correlation values obtained at different distances as evidence of structural linearity along that orientation. Cumulative correlation ($I_{C}$) is defined by

$$I_{C}(d)=\overset{d_{x}}{\underset{d=1}{\sum }}C(d)\Delta d,$$ (4)

where d is the distance and C(d) is the correlation value at distance d.

As the cumulative correlation increases with each additional GLCM distance, the difference between consecutive values gradually decreases. To minimize noise, a distance threshold (dx) was defined to determine the point at which sufficient evidence of linearity was achieved. Specifically, the threshold distance was identified as the point where the fractional difference between consecutive cumulative correlation values ($I_{C}$) fell below 10%. A cumulative correlation value is calculated for each direction (0 deg, 45 deg, 90 deg, and 135 deg). The principal angle is then calculated using Eq. (5) and is defined as the angle at which the largest number of structures are aligned, having the largest $I_{Cd_{x}}$ value.

$$\theta^{*}=\arg_{\theta \in X}\;\max\;I_{Cd_{x}}(\theta ),\;X=(0,45,90,135\;\deg).$$ (5)

The dominant orientation angle of collagen fibers was determined by rotating the image from −22  deg to +22  deg around the principal angle and computing the cumulative correlation at each rotation. Image rotation was performed using the *imrotate* function in MATLAB. A masking-based approach was applied to ensure that only pixels within the original image domain were included in the GLCM calculations at each rotation angle.

$$\sigma_{I_{c}}=\sqrt{\frac{1}{44}\overset{\theta^{*}+22}{\underset{\theta =\theta^{*}-22}{\sum }}[I_{c_{dx}}(\theta )-\overset{¯}{I_{c_{dx}}}(\theta ){]}^{2}}\;\text{where}\;\overset{¯}{I_{c_{dx}}}(\theta )=\frac{1}{45}\overset{\theta^{*}+22}{\underset{\theta =\theta^{*}-22}{\sum }}I_{c_{dx}}(\theta ).$$ (6)

Finally, the standard deviation of the cumulative correlation values computed at each rotated angle around the principal orientation was calculated to obtain a single quantitative metric, as defined in Eq. (6). Given the established relationship between correlation and structural linearity, this standard deviation serves as an estimate of collagen fiber orderliness across the evaluated orientations.

#### 2.5.5 Windowed Hough transform

Hough Transform is an established technique for detecting lines and other geometric shapes in images.^43^ It has been previously applied to evaluate collagen fiber linearity. Most prior studies, however, compute the Hough transform across entire FOVs.^32^^,^^44^ When the goal is to assess the linearity of individual collagen bundles, whole-FOV analysis can yield misleading results. For instance, SHG images of normal thyroid follicles often exhibit polygonal follicular structures, and evaluating linearity over the whole FOV may incorrectly suggest nonlinear organization, even though individual collagen bundles remain locally linear. To overcome this limitation, we compute local Hough transforms by subdividing the FOV into smaller regions, enabling more accurate quantification of collagen linearity at the bundle level.

**Fig. 1:**
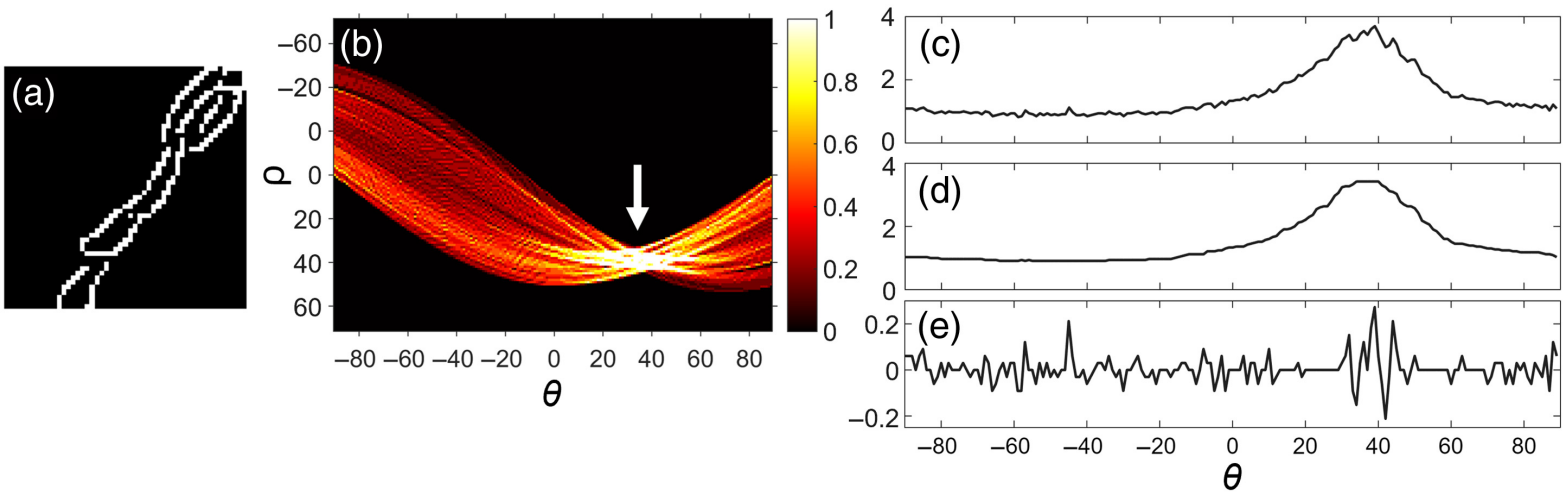
Example of windowed Hough transform analysis. (a) A 51×51  pixels edge-detected window with white indicating logical 1 (edge pixels) and black indicating logical 0. A predominant linearity of edge pixels is visible. (b) Resulting Hough transform with a concentration of binned intersections at ∼35  deg in θ (white arrow). (c) Summation of the Hough transform along θ. (d) Median filtered summation. (e) Difference between the filtered summation and the original summation.

In summary, edge detection is first performed within each selected image subsection. For every detected edge point, imaginary lines are drawn and represented in polar coordinates by ρ and θ, where ρ denotes the perpendicular distance from the origin to the line, and θ represents the angle between the x-axis and the line. These lines are plotted in Hough space as ρ versus θ, producing a sinusoidal curve for each line drawn through an edge-detected point. Because every edge point generates its own sinusoid, the resulting Hough space typically contains many overlapping curves in the ρ−θ plot [Fig. 1(b)]. Intersections of these sinusoidal curves (indicated by a white arrow) correspond to lines that pass through multiple edge-detected points in the original image. A greater number of intersections indicates the presence of a more prominent linear edge. These intersections are then counted and binned at specific (θ,ρ) coordinates in Hough space. Furthermore, parallel lines in the image produce clusters of intersections sharing similar θ values but differing ρ. In highly linear images [Fig. 1(a)], the resulting Hough transform displays a strong concentration at a single dominant θ value across a range of ρ [Fig. 1(b)], reflecting the coherent orientation of collagen bundles.

In this study, the windowed Hough transform was implemented using the *hough* function in MATLAB. After preprocessing, Canny edge detection was applied to each FOV, producing a 512×512 binary matrix. A sliding window of 51×51  pixels was then moved across the FOV, allowing the Hough transform to be computed locally within each subsection. For each θ, the sum of the top 5 binned values along ρ was calculated and plotted. Windows exhibiting low linearity produced more broadly distributed binned values across θ, appearing as “noise” due to numerous sinusoidal intersections lacking a dominant orientation. To quantify this, the variation in the summation plot [Fig. 1(e)] was obtained by subtracting a median-filtered version [Fig. 1(d)] from the original plot [Fig. 1(c)]. The standard deviation of this variation was defined as the Hough standard deviation (Hough SD) and assigned to the center pixel of the window. The window was then shifted by 10 pixels, and the process was repeated over the entire FOV. The average of all Hough SDs across the FOV was computed to yield a single Hough SD value representative of that FOV. A higher Hough SD indicates greater variability in the detected edge angles, corresponding to the presence of collagen fibers oriented in multiple directions, that is, nonlinear alignment. Conversely, a lower Hough SD reflects less angular variability, suggesting stronger local alignment and more linear orientation of collagen fibers within the analyzed window. The window size of 51×51  pixels was selected after evaluating multiple candidate sizes. The Hough SD plateaued at ∼51×51  pixels, beyond which a consistent increase in SD was observed. This increase did not appear to arise from meaningful changes in fiber orientation, but rather from the inclusion of additional structures within larger windows, potentially inflating the angular dispersion.

#### 2.5.6 Fourier transform and power spectral density analysis

Fourier transform analysis has been widely employed to characterize frequency patterns within collagen structures using SHG imaging data.^45^^,^^46^ To compare the spatial frequency distribution of collagen organization between normal and cancerous tissue samples, a two-dimensional Fast Fourier Transform (2D FFT) was computed for each image. The corresponding power spectrum was obtained as the squared magnitude of the FFT. A radial frequency profile was then derived to quantify the distribution of power as a function of spatial frequency, effectively grouping FFT data into bands corresponding to progressively higher frequencies. The radial distance matrix was computed using the following equation:

$$R(x,y)=\sqrt{(x-\frac{M}{2}{)}^{2}+(y-\frac{N}{2}{)}^{2}},$$ (7)

where R is the radial distance and M and N are image dimensions. For each radial distance R, the power at all pixels within the radial band was averaged:

$$\text{Radial Profile}(r)=\frac{\sum_{(x,y)\in r}P(x,y)}{\text{Count of pixels in}\;r},$$ (8)

where P is the power at coordinates (x,y). Several quantitative metrics were calculated based on the Fourier transform spectral density analysis including

1. *Power spectral density (PSD):* The total energy in the frequency domain was computed as
  $$\text{Total Power}=\overset{M-1}{\underset{u=0}{\sum }}\overset{N-1}{\underset{v=0}{\sum }}P(u,v).$$ (9)

Apart from the total power, to investigate specific frequency ranges, the power spectrum was divided into three bands based on normalized radial distances relative to the maximum radius $R_{\max}$.^16^ Low frequency was defined as values less than 0.2 times $R_{\max}$, mid frequency was defined as values greater than 0.2 and less than or equal to 0.7 times $R_{\max}$, and high frequency was defined as values greater than or equal to 0.7 times $R_{\max}$. The power within each band was summed to represent the low-, mid-, and high-frequency components, respectively. To account for potential power contributions beyond the defined radial range, any power outside $R_{\max}$ was incorporated into the high-frequency band. The specific cutoffs at 0.2 and 0.7 $R_{\max}$ were selected based on (i) empirical inspection of averaged radial spectra, which showed consistent transitions in power distribution near these normalized radii, and (ii) the need to isolate the high-frequency tail, which is more susceptible to noise, while retaining sufficient signal for statistical analysis. These thresholds therefore represent biologically interpretable and statistically stable boundaries rather than arbitrary divisions.

2. *Disorganization parameter:* Shannon entropy is used as a measure of the uncertainty or randomness in the frequency distribution of the collagen fiber FFT image. This quantifies the amount of disorder or complexity in the spatial frequency content of the images, and it is a common tool to quantify the degree of information present in a signal:
  $$\text{Entropy}=-\overset{R_{\max}}{\underset{r=1}{\sum }}p(r)\log(p(r)+\epsilon ).$$ (10)

Here, p(r) is the normalized radial profile:

$$p(r)=\frac{\text{Radial Profile}(r)}{\sum_{r=1}^{R_{\max}}\text{Radial Profile}(r)}.$$ (11)

From this metric, the disorganization parameter (DP) can be calculated; a higher DP indicates more disorder within the FFT, correlating to less organization in the spatial domain.

3. *Relative entropy (Kullback-Leibler Divergence):* Kullback-Leibler (KL) divergence, also known as “relative entropy,” quantifies the “distance” that a data set, in this case the PSD of an FFT image, deviates from a uniform distribution.^18^ Higher relative entropy indicates a higher level of deviance, indicating higher variability and lower uniformity. It is given by the KL divergence:
  $$H_{\mathrm{rel}}=\overset{N}{\underset{i=1}{\sum }}P(i)\log(\frac{P(i)}{Q(i)})+\epsilon ,$$ (12)
  where P(i) is the normalized PSD at frequency i, Q(i) is the uniform distribution matrix of ones equal in dimension to P(i), and ϵ is a small constant to avoid logarithms of zero.
4. *Weighted frequency center (WFC):* The weighted frequency center gives an average frequency weighted by the radial power distribution in the frequency domain. This approach quantifies the low-frequency signal versus the high-frequency signal with respect to PSD, comparing data using discrete, radially averaged frequency bands along the power spectrum. Low WFC indicates more low-frequency content with smoother structures, whereas high WFC suggests the presence of finer textures or patterns. It is computed as
  $$\mathrm{WFC}=\frac{\sum_{r=1}^{N}f(r)\cdot \text{Radial Profile}(r)}{\sum_{r=1}^{N}\text{Radial Profile}(r)},$$ (13)
  where f(r) is the frequency at radial distance r, radial profile(r) is the power in the frequency band around radial distance r, computed from the FFT power spectrum, and N is the number of radial bins.

#### 2.5.7 Anisotropy parameter

To assess anisotropy of the collagen in an entire FOV, we apply a method as described in Hall et al.^47^ Briefly, for each FOV, a 2D FFT was performed to transform the image into spatial frequency space. The resulting spectrum was integrated along polar coordinates to determine the angular distribution of spatial frequencies, reflecting how strongly pixel intensities in the original image favor particular orientations and thereby indicating collagen directionality. The anisotropy parameter was then calculated as a normalized phasor sum of this angular distribution, representing the average direction and magnitude of the 2D FFT. Values approaching 0 indicate isotropy, corresponding to random or disorganized collagen structures, whereas values approaching 1 indicate anisotropy, reflecting a more organized and aligned fiber arrangement.

#### 2.5.8 Windowed anisotropy parameter

As discussed for the Hough transform, one challenge in analyzing our sample FOVs is the presence of multiple follicles within a single image. When averaged across the entire FOV, this heterogeneity can give the appearance of disorganized fibers oriented in multiple directions. To address this, the anisotropy parameter analysis was adapted to a sliding-window approach, similar to the windowed Hough transform. The same window parameters were used, 51×51  pixel windows with a 10-pixel step size. For each window, a 2D FFT was computed, and the angular distribution was determined as described previously for the whole-FOV anisotropy calculation.

### 2.6 Categorization of Quantitative Metrics

To systematically characterize the collagen remodeling in PTC, the 35 quantitative metrics were grouped into four functional families based on their mathematical formulation and biological interpretation:

#### 2.6.1 Geometric and directional metrics

This family includes F/B ratio, Hough SD, whole FOV anisotropy, and windowed anisotropy. These metrics quantify the spatial orientation and alignment of collagen fibers at multiple scales.

#### 2.6.2 Textural metrics

This family includes cluster tendency, cluster shade, cluster prominence, homogeneity, contrast, dissimilarity, inverse variance, inverse difference normalized, inverse difference moment normalized, autocorrelation, correlation, energy, maximum probability, entropy, difference entropy, difference variance, sum entropy, sum average, sum of squares variance, information measure of correlation 1, information measure of correlation 2, and OD-GLCM. These features are primarily derived from GLCM analysis and clustering statistics. They characterize the local spatial relationships between pixel intensities and quantify texture heterogeneity.

#### 2.6.3 Intensity and content metrics

This category includes CAF and CAF FB. These metrics reflect the overall magnitude or abundance of the SHG signal and provide an estimate of collagen content.

#### 2.6.4 Frequency-domain metrics

This family includes total power, low-frequency power, mid-frequency power, high-frequency power, weighted frequency center, disorganization parameter, and relative entropy. Derived from Fourier transform and spectral analysis, these metrics characterize the dominant spatial frequencies and structural periodicity of collagen fibers. They provide insight into whether the collagen architecture is dominated by thick, mature bundles (low-frequency components) or finer, newly synthesized fibrillar structures (higher-frequency components).

## 3 Results

**Fig. 2:**
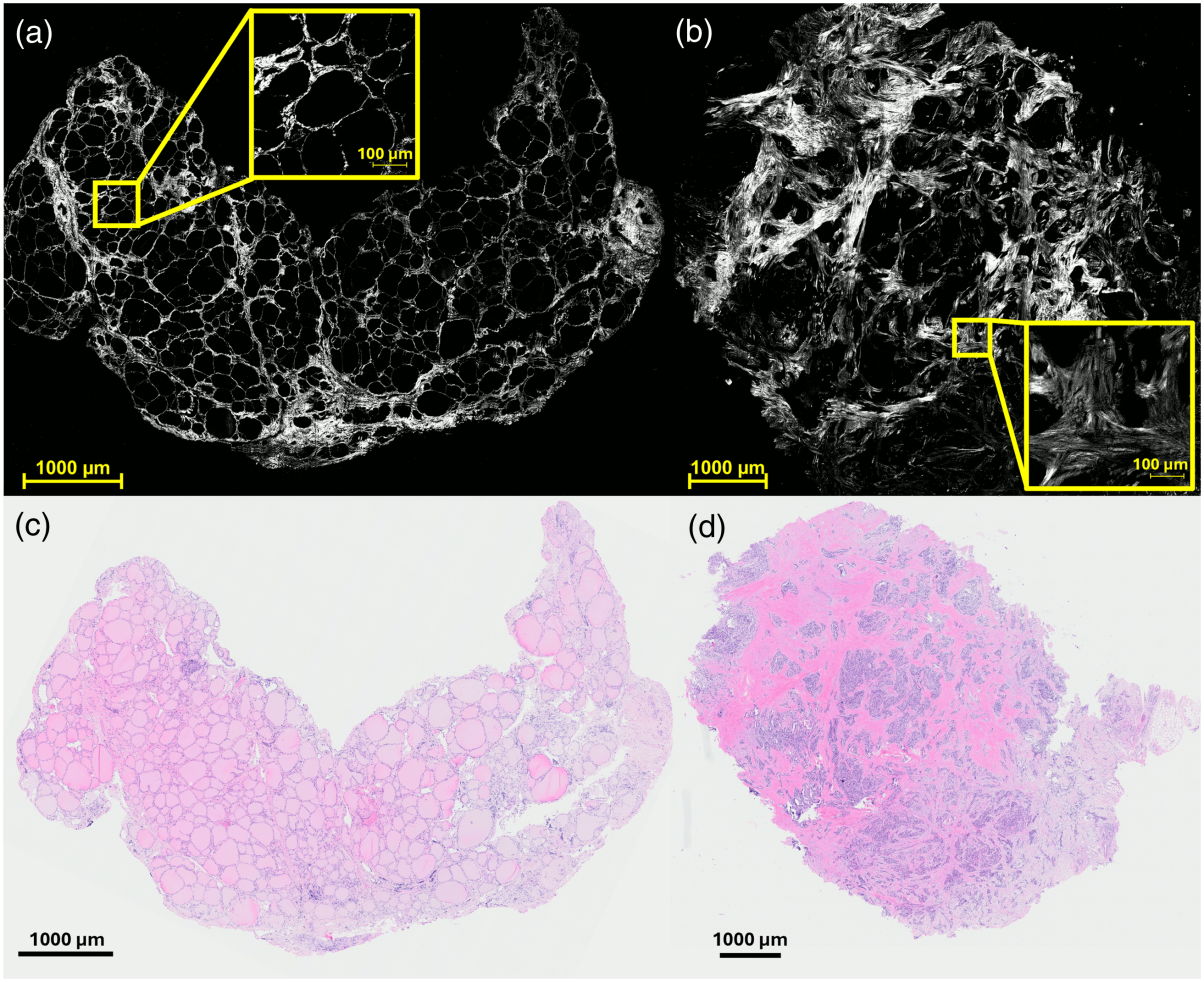
MIPs of backward detected whole-slide SHG images and corresponding H&E images of (a, c) normal and (b, d) cancerous human thyroid tissue.

Figures 2(a) and 2(b) show representative whole-slide SHG images, whereas Figs. 2(c) and 2(d) show corresponding H&E images of normal and cancerous thyroid tissues respectively. Whole-slide SHG reconstructions were generated by tiling the MIPs from individual FOVs. To correct for illumination inconsistencies such as peripheral darkening introduced by the optical system, BaSiC^48^ shading correction was applied to each MIP. The corrected images were then stitched together using the 5% overlap applied during acquisition, producing the composite whole-slide images shown in Fig. 2.

In normal thyroid parenchyma, the thyroid follicles contain colloid and are lined by thyroid follicular cells with round to oval, basally located nuclei and eosinophilic cytoplasm. The surrounding stroma consists of thin, delicate fibrous septa that separate thyroid lobules and contain extracellular structural proteins, including collagen.^49^^,^^50^ On H&E staining, the eosin component highlights these extracellular structural proteins in pink. Accordingly, the H&E image of normal thyroid tissue [Fig. 2(c)] demonstrates thyroid follicles embedded within this delicate and organized collagen-containing stroma. This is consistent with the corresponding SHG image [Fig. 2(a)], which shows well-organized collagen surrounding intact thyroid follicles.

By contrast, thyroid malignancies such as PTC are characterized by parenchymal overgrowth and increased stromal fibrosis and collagen deposition. This leads to marked disruption of the normal follicular architecture. Although fibrotic inflammatory conditions may also exhibit increased fibrosis and collagen deposition, the follicular architecture is generally preserved.^51^ In thyroid cancer, however, the normal uniform follicular structures are obliterated and replaced by infiltrating tumor cells and dense, irregular collagen bands,^52^ as observed in Fig. 2(d). This finding corroborates the corresponding SHG image [Fig. 2(b)], which demonstrates disorganized collagen and loss of follicular structure in cancerous thyroid tissue.

Although Fig. 2 illustrates qualitative differences in the whole-slide SHG images, all quantitative analyses were performed on randomly imaged FOVs, as described in Secs. 2.2 and 2.3. This approach was intentionally adopted to align with our ultimate goal of clinical translation.

**Fig. 3:**
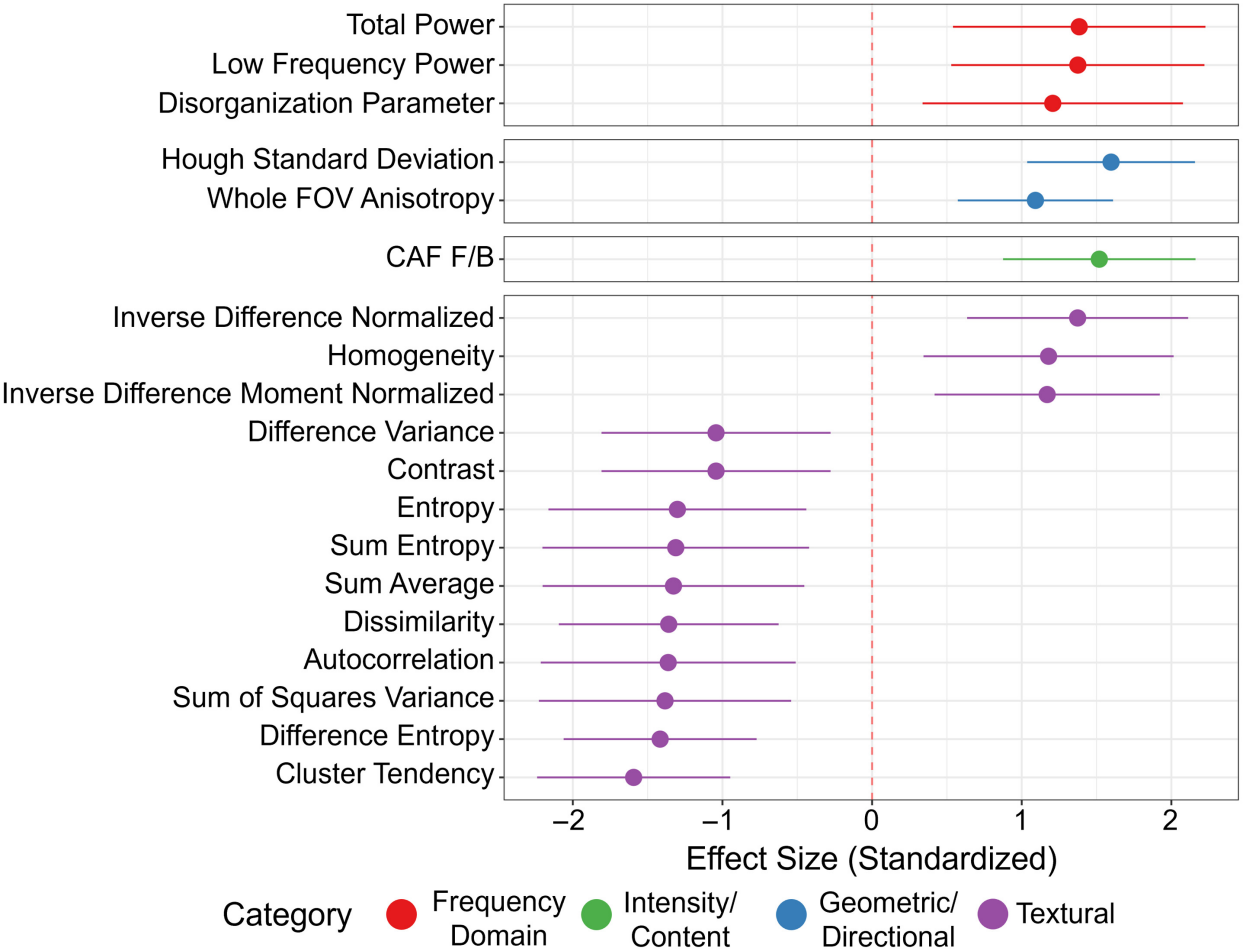
Effect sizes of quantitative metrics showing a statistically significant difference between groups after FDR.

To rigorously evaluate collagen remodeling in PTC, we applied LMM across 35 quantitative SHG-derived metrics. After FDR adjustment for multiple comparisons, 19 of 35 metrics remained statistically significant (FDR-adjusted P<0.05). Standardized effect sizes ranged from moderate to very large magnitude, with several metrics exceeding |d|>1.5 and multiple features demonstrating |d|>1.0. These large effect sizes indicate substantial separation between normal and cancer tissues across multiple structural domains of collagen organization. Figure 3 shows a forest plot depicting effect sizes of the different metrics that showed statistical significance after FDR.

Table 2 summarizes the performance of all metrics. It provides information on the category, effect size, P value before and after FDR, and the ICC.

**Table 2:** Performance summary of all 35 metrics grouped into their respective categories.

Metric	Category	Effect (d [95% CI])	P value	FDR-adjusted P value	ICC
**Total power**	FD	1.38 [0.54, 2.23]	0.0108	0.0390	0.6821
**Low-frequency power**	FD	1.37 [0.53, 2.22]	0.0113	0.0390	0.6767
**DP**	FD	1.21 [0.34, 2.08]	0.0235	0.0467	0.5738
**Relative entropy**	FD	1.21 [0.26, 2.15]	0.0338	0.0563	0.7033
**WFC**	FD	−1.18 [−2.14, −0.22]	0.0379	0.0602	0.7031
**Mid-frequency power**	FD	0.98 [0.13, 1.83]	0.0485	0.0738	0.4246
**High-frequency power**	FD	0.72 [−0.22, 1.66]	0.1609	0.2011	0.4557
**CAF F/B**	I/C	1.52 [0.88, 2.16]	0.0014	0.0168	0.4518
**CAF**	I/C	0.53 [−0.68, 1.74]	0.4056	0.4437	0.7871
**Hough SD**	G/D	1.60 [1.04, 2.16]	0.0004	0.0153	0.3864
**Anisotropy**	G/D	1.09 [0.57, 1.61]	0.0029	0.0200	0.0403
**Windowed anisotropy**	G/D	0.93 [0.08, 1.78]	0.0584	0.0851	0.3984
**F/B**	G/D	−0.85 [−1.83, 0.13]	0.1198	0.1612	0.5596
**Cluster tendency**	T	−1.59 [−2.24, −0.95]	0.0011	0.0168	0.5600
**Difference entropy**	T	−1.42 [−2.06, −0.77]	0.0022	0.0195	0.3556
**Inverse difference normalized**	T	1.37 [0.63, 2.11]	0.0057	0.0291	0.4810
**Dissimilarity**	T	−1.36 [−2.09, −0.62]	0.0058	0.0291	0.4614
**Sum of squares variance**	T	−1.38 [−2.23, −0.54]	0.0108	0.0390	0.6803
**Autocorrelation**	T	−1.36 [−2.21, −0.51]	0.0123	0.0390	0.6747
**Inverse difference moment normalized**	T	1.17 [0.42, 1.92]	0.0140	0.0402	0.3671
**Sum average**	T	−1.33 [−2.20, −0.45]	0.0157	0.0402	0.6803
**Entropy**	T	−1.30 [−2.16, −0.44]	0.0161	0.0402	0.6332
**Sum entropy**	T	−1.31 [−2.20, −0.42]	0.0180	0.0421	0.6953
**Homogeneity**	T	1.18 [0.34, 2.02]	0.0218	0.0467	0.4999
**Contrast**	T	−1.04 [−1.81, −0.28]	0.0253	0.0467	0.3290
**Difference variance**	T	−1.04 [−1.81, −0.28]	0.0253	0.0467	0.3290
**Inverse variance**	T	−1.14 [−2.02, −0.26]	0.0315	0.0552	0.5442
**Information measure of correlation 2**	T	−0.93 [−1.96, 0.10]	0.1059	0.1483	0.6624
**OD-GLCM**	T	0.40 [−0.16, 0.96]	0.1595	0.2011	0.0000
**Correlation**	T	−0.66 [−1.57, 0.25]	0.1844	0.2226	0.4114
**Energy**	T	0.76 [−0.40, 1.92]	0.2233	0.2605	0.7850
**Maximum probability**	T	0.67 [−0.50, 1.84]	0.2818	0.3182	0.7639
**Information measure of correlation 1**	T	0.22 [−0.63, 1.06]	0.6185	0.6560	0.2716
**Cluster shade**	T	−0.19 [−1.22, 0.84]	0.7234	0.7447	0.4962
**Cluster prominence**	T	0.04 [−0.94, 1.03]	0.9335	0.9335	0.4310

FD, frequency domain; I/C, intensity/contrast; G/D, geometric/directional; T, textural; CI, confidence intervals; ICC, interclass correlation coefficient.

### 3.1 Loss of Geometric Organization

**Fig. 4:**
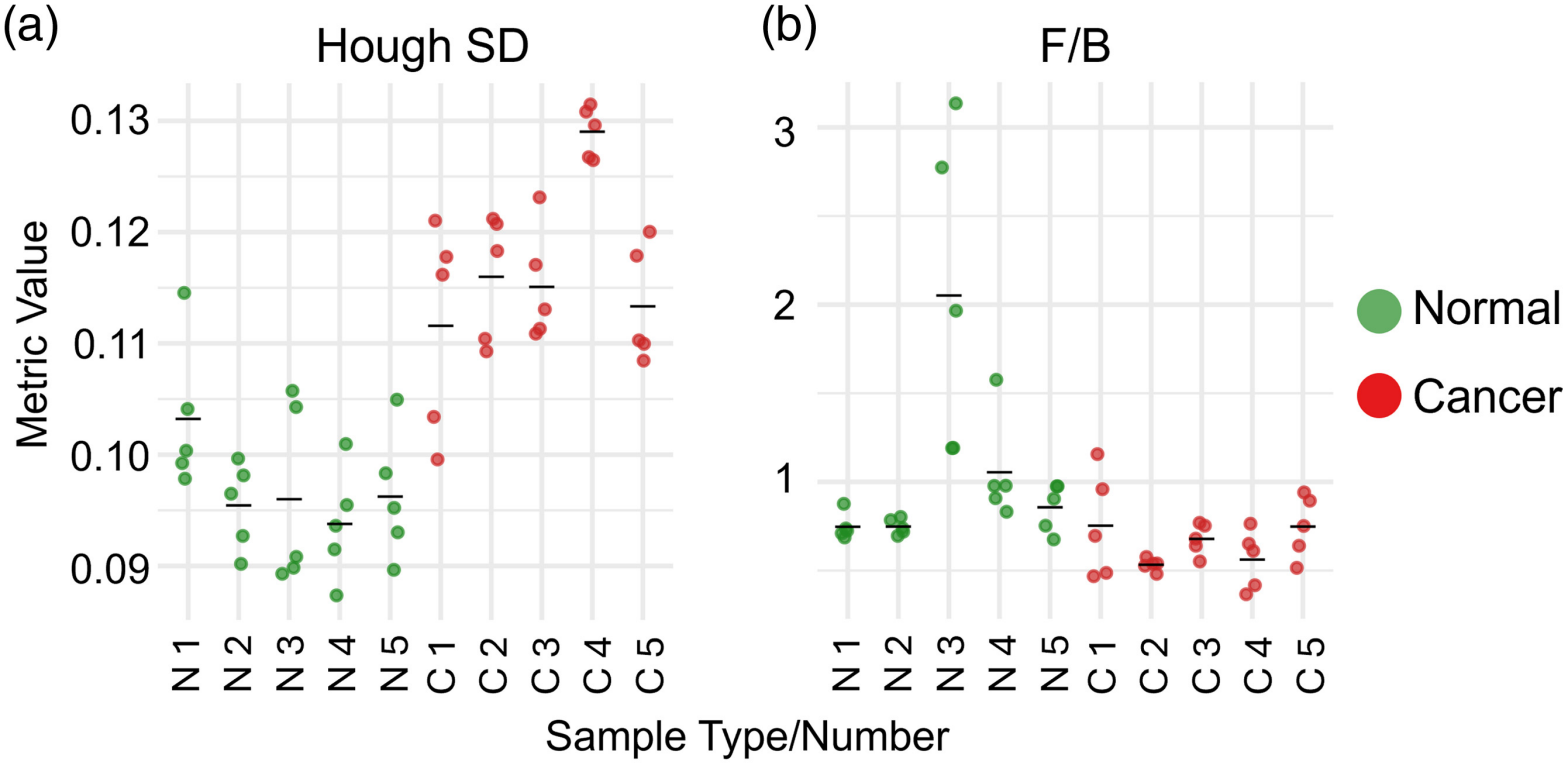
Patient-level distribution of two representative geometric/directional metrics. (a) Hough SD, a statistically significant metric (FDR-adjusted P=0.015). (b) F/B ratio, which did not show statistical significance between groups (FDR-adjusted P=0.162). Each colored dot represents an individual FOV. Five FOVs were acquired per sample; therefore, each independent sample contributes five data points to the plot. N = normal, C = cancer. The black bar indicates the mean value for each group.

Geometric and directional metrics demonstrated some of the strongest discriminative powers. Hough SD emerged as the most significant geometric biomarker (FDR = 0.015, d=1.60, CI = [1.04, 2.16]), indicating markedly increased orientation dispersion of collagen fibers in PTC. Whole FOV anisotropy (FDR = 0.020, d=1.09, CI = [0.57, 1.61]) was also significantly elevated in cancer samples. Windowed anisotropy and F/B ratio did not show statistical significance after FDR adjustment. However, both metrics demonstrated higher effect sizes than 0.8 (|d|=0.93 and 0.85, respectively), suggesting that the lack of statistical significance may be attributable to limited sample size rather than the absence of a meaningful group difference. Figure 4 presents patient-level distribution data for two representative metrics: one that demonstrated a statistically significant difference between groups (Hough SD) and one that did not remain statistically significant (F/B ratio) after FDR adjustment. Unlike the case of Hough SD, a clear overlap can be seen between the normal and cancer samples in the plot depicting the F/B ratio spread.

Importantly, geometric metrics exhibited moderate intraclass correlation (e.g., ICC = 0.39 for Hough SD), indicating appreciable within-patient spatial heterogeneity. Despite this, the group level difference remained statistically significant. This suggests that while cancer induces strong directional disruption, the remodeling process remains locally variable across FOVs.

### 3.2 Disruption of Textural Patterns and Clustering

Textural analysis revealed widespread alterations in local collagen architecture. Cluster Tendency demonstrated a pronounced reduction in PTC (FDR = 0.017, d=−1.59, CI=[−2.24,−0.95]), consistent with increased disorganized collagen. Significant decreases were also observed for difference entropy (FDR = 0.019, d=−1.42, CI=[−2.06,−0.77]), dissimilarity (FDR = 0.029, d=−1.36, CI=[−2.09,−0.62]), sum of squares variance, autocorrelation, sum average, entropy, and sum entropy (all p<0.05, |d|≈1.3−1.4). Conversely, metrics reflecting inverse or normalized directional smoothness such as inverse difference normalized (FDR = 0.029, d=1.37, CI = [0.63, 2.11]) and inverse difference moment normalized (FDR = 0.040, d=1.17, CI = [0.42, 1.92]) were significantly elevated, further supporting structural irregularity. Homogeneity (FDR = 0.047, d=1.18, CI = [0.34, 2.02]), contrast (FDR = 0.047, d=−1.04, CI=[−1.81,−0.28]), and difference variance (FDR = 0.047, d=−1.04, CI=[−1.81,−0.28]) also reached significance. Similar to the geometric/directional metrics, a few of the textural metrics that did not reach statistical significance exhibited effect sizes approaching or slightly exceeding |d|=0.8. This pattern may indicate limited statistical power and suggests that the absence of significance could be related to sample size rather than a lack of underlying group differences.

**Fig. 5:**
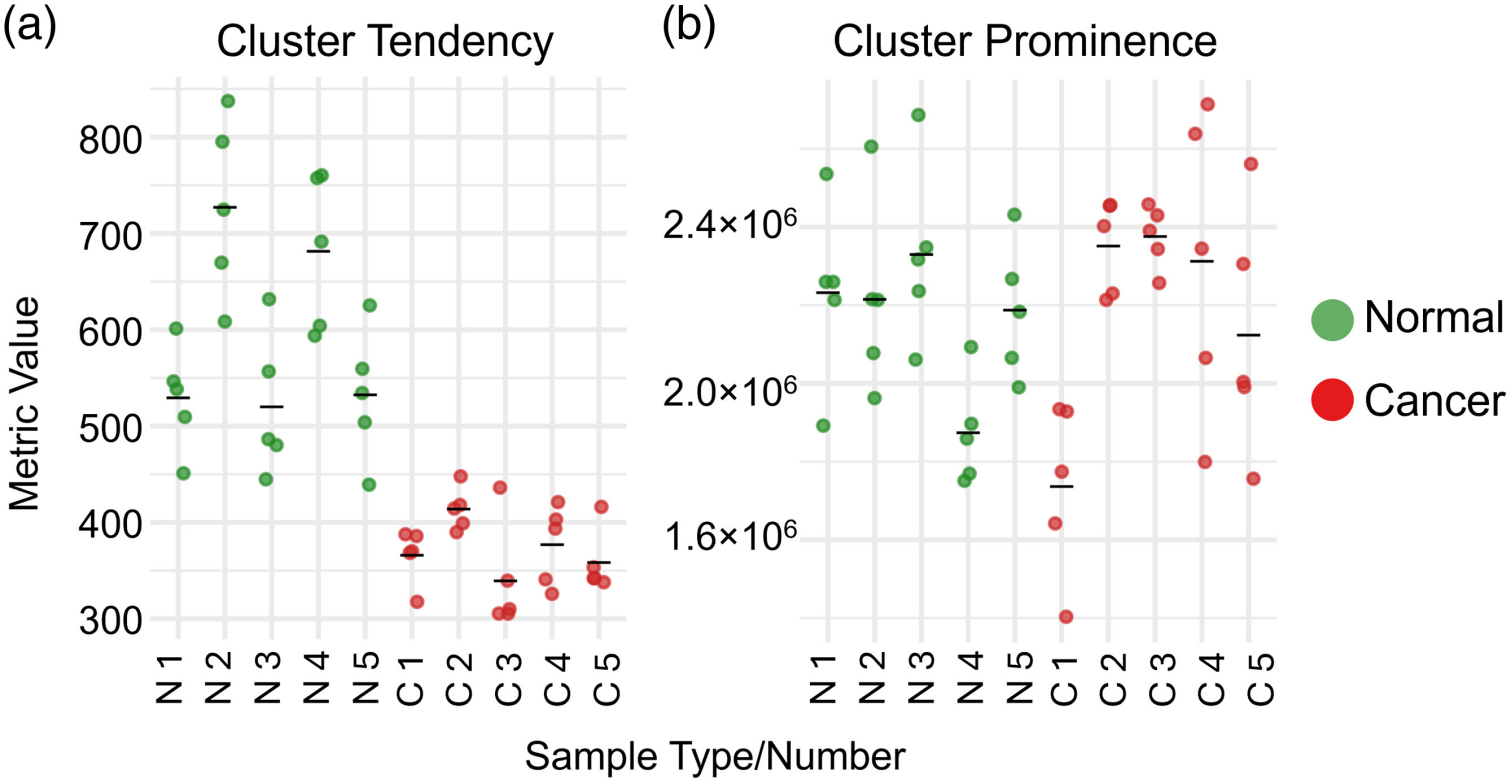
Patient-level distribution of two representative texture metrics. (a) Cluster tendency, a statistically significant metric (FDR-adjusted P=0.017). (b) cluster prominence, which did not show statistical significance between groups (FDR-adjusted P=0.933). Each colored dot represents an individual FOV. Five FOVs were acquired per sample; therefore, each independent sample contributes five data points to the plot. N = normal, C = cancer. The black bar indicates the mean value for each group.

Figure 5 presents patient-level distribution data for two representative metrics: one that demonstrated a statistically significant difference between groups (cluster tendency) and one that was not statistically significant (cluster prominence) before and after FDR correction.

Collectively, these results indicate that PTC is characterized by substantial degradation of organized collagen texture and loss of spatial regularity.

### 3.3 Alterations in Collagen Content and Frequency Distribution

CAF F/B ratio (FDR = 0.017, d=1.52, CI = [0.88, 2.16]) and total power (FDR = 0.039, d=1.38, CI = [0.54, 2.23]) were significantly increased in PTC, consistent with enhanced collagen deposition and remodeling. Notably, some of these metrics demonstrated high ICC values (>0.68), indicating that collagen accumulation represents a relatively stable, global feature across patient specimens.

Disorganization parameter (FDR = 0.047, d=1.21, CI = [0.34, 2.08]) and low-frequency power (FDR = 0.039, d=1.37, CI = [0.53, 2.22]) were also significant and showed an increase in cancer samples. Mid-frequency power, relative entropy, and weighted frequency center did not survive multiple-comparison correction. This indicates that although spectral redistribution trends were observed, only low-frequency power and disorganization parameter demonstrated statistically robust evidence after controlling for multiple testing. However, effect sizes of all the aforementioned metrics that did not show significance were close to or slightly greater than |d|=0.8, indicating that the absence of significance could be related to sample size rather than a lack of underlying group difference.

**Fig. 6:**
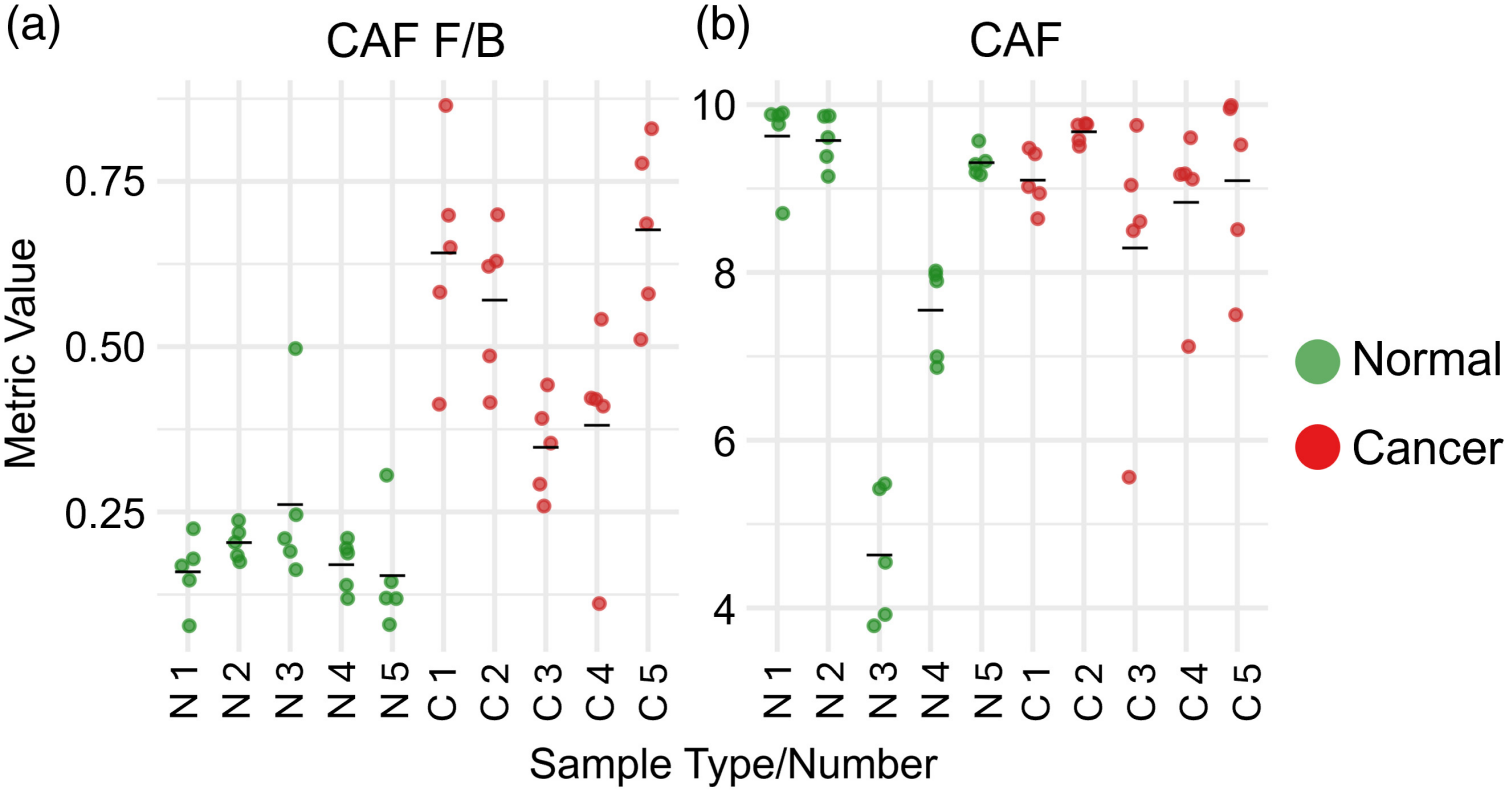
Patient-level distribution of two representative intensity metrics. (a) CAF F/B, a statistically significant metric (FDR-adjusted P=0.017). (b) CAF, which did not show statistical significance between groups (FDR-adjusted P=0.444). Each colored dot represents an individual FOV. Five FOVs were acquired per sample; therefore, each independent sample contributes five data points to the plot. N, normal; C, cancer. The black bar indicates the mean value for each group.

Figure 6 presents patient-level distribution data for two representative metrics: one that demonstrated a statistically significant difference between groups (CAF F/B) and one that did not remain statistically significant (CAF) after FDR correction.

## 4 Discussion

In this study, we used SHG microscopy to successfully differentiate between normal and cancerous human thyroid tissue. Out of a total of 35 quantitative metrics used, 19 metrics demonstrated statistically significant differences between the groups. Of the remaining 16, 5 metrics demonstrated consistent directional trends, and 5 did not display any differences. The metrics exhibiting directional trends showed consistent differences between groups, with mean values persistently higher or lower in one group compared to the other. Although these metrics did not reach statistical significance, they suggest the presence of underlying biological trends.

Because reporting standardized effect sizes alongside P values presented a clearer quantification of the magnitude and precision of the biological differences between cancer and normal tissues, effect sizes have been reported along with the corresponding 95% confidence intervals. This helped reduce interpretational limitations and provided more analytically robust metrics on which to base our conclusions. This analysis revealed that several metrics that did not show statistical significance exhibited effect sizes approaching or slightly exceeding |d|=0.8. These findings suggest that the lack of statistical significance could be attributed to a limited sample size rather than the absence of a meaningful group difference or biological trend. Increasing the number of samples would enhance statistical power, potentially yielding significant results. Nonetheless, the consistent directional shifts observed across these metrics indicate their potential utility in differentiating normal and cancerous thyroid tissues based on SHG-derived collagen features.

The primary goal of this study was to distinguish between normal and cancerous samples. Hence, 2D MIP images were used for quantification. Although, in principle, this approach may introduce bias in texture, orientation, and spatial correlation metrics, our objective was not to characterize or quantify the absolute 3D collagen architecture of each group. Identical image processing and MIP procedures were applied to all samples in both the normal and cancerous groups, ensuring that the extracted metrics reflect relative differences between groups, aligning with the overall goal of the study. That said, prior studies^53^ have demonstrated that applying GLCM-based texture analysis to three-dimensional SHG datasets preserves axial information and enables a more comprehensive characterization of collagen architecture. A potential future direction of this work is to incorporate 3D analysis to detect subtle differences in collagen microstructure, particularly when distinguishing among different subtypes of thyroid cancer.

Along similar lines, this study focuses exclusively on backward-detected SHG signals to prioritize *in vivo* applicability and clinical translation, where forward-detected signal acquisition is not feasible. Although inclusion of forward-detected images could provide additional structural information, such data would have limited relevance to the intended clinical use case. Consequently, restricting the analysis to backward-detected signals ensures alignment with translational goals. A limitation of this approach is that potential differences observable in forward-detected signals are not explored. Nevertheless, our results demonstrate consistent, statistically significant quantitative differences between normal and cancerous groups using imaging configurations that are directly compatible with clinical implementation.

Hough SD quantifies the local linearity and organization of collagen fibers, with higher values reflecting increased fiber disorganization and intersection. Cancer samples exhibited elevated Hough SD compared with normal thyroid tissues, indicating greater local collagen disarray. Likewise, the clustering tendency metric assesses the spatial aggregation of similar gray-level intensities. Its reduction in cancer samples suggests increased heterogeneity in collagen SHG signal, consistent with the irregular and disorganized extracellular collagen architecture characteristic of thyroid carcinoma.

**Fig. 7:**
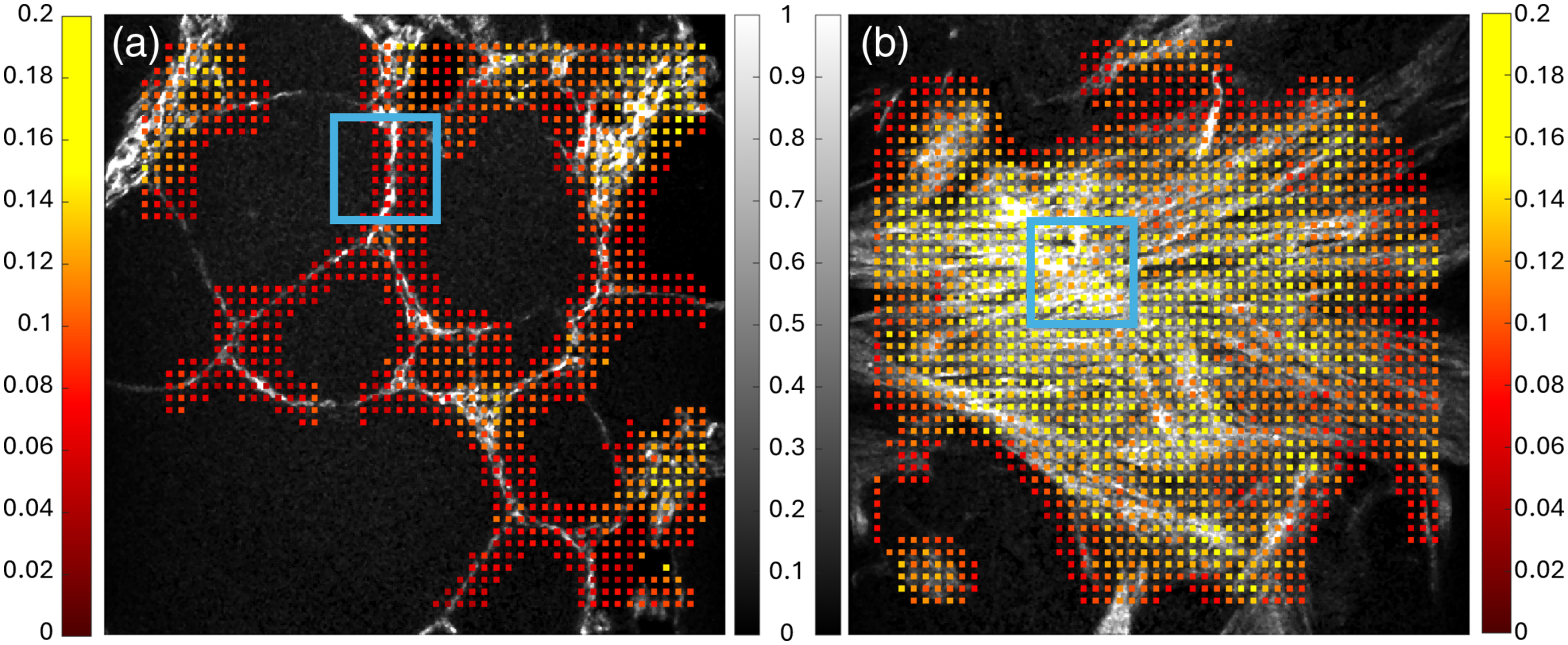
Representative grayscale SHG MIPs of normal thyroid (a) and cancer (b) overlaid with corresponding heatmaps of Hough SD values. The color bar from dark red to yellow represents Hough SD values. The grayscale color bar represents the SHG image. The blue box in panel (a) depicts an area with low Hough SD or relatively linear or organized collagen, and the blue box in panel (b) depicts an area with high Hough SD or disorganized collagen. Both MIPs are 512×512  pixels or about 481  μm×481  μm.

As seen in Fig. 7, the Hough SD is lower in areas of very linear and organized collagen [such as the boxed area of normal thyroid in Fig. 7(a)] and higher in areas of much more disorganized collagen [such as the blue boxed area in Fig. 7(b)]. The average Hough SD of the FOV represented in Fig. 7(a) was 0.0899, and the cluster tendency of the normal FOV was 556.7. On the contrary, the average Hough SD of the cancer FOV represented in Fig. 7(b) was 0.1267 and the cluster tendency of the FOV was 393.6.

GLCM can be computationally expensive but did produce various metrics with statistical significance, in addition to more metrics that showed directionality but no significance. One limitation regarding the GLCM-based methods in our work is that we have not fully addressed the inherent variability embedded in the creation of the GLCM matrix. As it stands right now, GLCM and OD-GLCM by their nature have quite a few modifiable parameters, such as the number of gray levels to increase granularity in the gray-level scaled image. These factors can affect algorithmic performance depending on the optical system and tissue type, potentially reducing reproducibility and generalizability. Further studies should mitigate variation caused by tunable parameters to standardize the method for broader applicability. One possible way could be integrating gray-level invariant GLCM metrics in the computation.^54^

For the Fourier transform-based feature analysis (categorized as frequency domain), total power, low-frequency power, and DP showed statistically significant differences between the two groups, and multiple parameters showed promising directionality but lacked significance. The significantly higher total power observed in cancerous tissue suggests a greater overall energy distribution in the frequency domain. This increase may reflect the disorganized and heterogeneous collagen architecture often associated with TMEs. Cancer progression involves extensive remodeling of the ECM, leading to increased signal intensity and structural complexity in SHG images. The power spectrum bands revealed that cancerous tissues exhibited elevated power across low, mid, and high frequencies. The higher power in low-frequency range reflects larger-scale collagen features, which may correspond to denser or thicker fibers. Increased power in the mid-frequency range may indicate alterations in the intermediate organization of collagen, such as branching patterns or irregular spacing between fibers. Elevated high-frequency power suggests a greater presence of fine structural details, such as fragmented or microfibrillar collagen. The lower WFC in cancerous tissues highlights a shift in the collagen structure toward larger bands relative to fine structures. The higher ratio underscores the structural heterogeneity characteristic of malignant tissues. The increased DP observed in cancerous tissues signifies greater randomness in the collagen fiber organization. A higher DP value indicates a less uniform and more complex collagen structure, consistent with the disordered ECM in tumor environments.

The anisotropy parameter describes normal and cancerous FOVs in an opposite manner compared with the Hough transform results. When applied to a full FOV capturing entire follicles, collagen surrounding normal thyroid follicles appears relatively isotropic, whereas cancer samples display thicker, more directionally aligned collagen bundles. However, this metric may be limited by highly disorganized and multidirectional collagen, also producing a low anisotropy parameter value. This means a low anisotropy parameter value could indicate either the very organized circular follicles of normal thyroid or highly disorganized collagen, which could represent cancer or other anatomical regions of the thyroid. Applying the anisotropy parameter to smaller windows helps overcome this limitation by restricting analysis to only portions of normal follicles at a time or small parts of thick collagen bundles in the case of cancer. At the FOV level, the anisotropy parameter primarily reflects differences in large-scale organization between follicular structures and collagen bundles. By contrast, at the window level, it is better suited to capturing local collagen disorganization. With this size restriction, the interpretation flips: single, small sections of normal thyroid follicle will appear highly anisotropic, whereas thick cancerous collagen bundles filling the entire window appear highly isotropic. Based on this, we expected the windowed anisotropy parameter to perform better than the whole FOV anisotropy. However, the results were reversed in this study, with the whole FOV having a lower P value and better separation than windowed anisotropy. One possible explanation is the markedly low intraclass correlation coefficient (ICC = 0.04) associated with the whole-FOV anisotropy metric, indicating substantial intrasample variability. This high intraclass variation may have influenced the statistical behavior of the metric. Nevertheless, additional analyses in larger cohorts will be necessary to further investigate the observed discrepancy between the two anisotropy measures and to clarify their relative performance.

A major limitation of this study is the small sample size, consisting of five normal and five cancerous thyroid specimens, each represented by five semirandom FOVs. Although increasing the number of samples and FOVs would likely enhance statistical power and reveal additional significant metrics, our intent was to evaluate algorithmic performance under data-limited conditions. This design reflects our ultimate goal of clinical translation to real-time *in vivo* or rapid *ex vivo* imaging, where only a few high-quality FOVs may be available and tissue quality may vary. Accordingly, we focused primarily on backward SHG signal and single MIP quantification rather than large composite images, emphasizing approaches most applicable to practical clinical imaging scenarios.

Several individual parameters did not reach statistical significance due to the limited sample size, and their specificity to distinct collagen characteristics could lead to inconclusive or misleading interpretations when considered in isolation. For example, Hough SD may suggest cancer-like disorganization in a normal region with atypical collagen, whereas other metrics may not capture such variation. A major strength of this study is the development and integration of a diverse set of quantitative metrics, minimizing dependence on any single parameter. In future work, these metrics will be incorporated into an automated analysis framework capable of comprehensively and unbiasedly characterizing relative changes in collagen architecture, thereby enhancing understanding of the local ECM in both normal and pathological tissues. The framework will incorporate feature selection methodologies to mitigate redundancy among highly collinear metrics, facilitating identification and prioritization of high-importance features through a systematic feature-ranking approach. Our future work will also focus on developing artificial intelligence-based algorithms for rapid and automated differentiation between normal and cancerous thyroid tissues. Although the current results and the proposed automated analysis framework provide a systematic and interpretable approach, comparing these methods with data-driven “black box” models will be essential to evaluate performance, generalizability, and clinical translation potential.

Although SHG microscopy provides valuable information on differences in collagen organization between normal and cancerous thyroid tissues, a comprehensive understanding of tissue biochemistry and additional markers of malignancy is essential. Future work will therefore employ multimodal nonlinear optical imaging to assess other tissue components, including lipids, elastin, and metabolic indicators such as NADH and FAD, which are known to change with malignancy. In addition to SHG, our imaging protocol will incorporate coherent anti-Stokes Raman scattering (CARS) microscopy and two-photon fluorescence (TPAF) microscopy to provide complementary biochemical and structural information.

Finally, this study focused exclusively on PTC, the most common subtype. For SHG-based optical biopsy to achieve broad clinical value, it must distinguish among follicular, oncocytic, medullary, anaplastic, and benign neoplastic subtypes. Our future work will expand the dataset to include a broader spectrum of thyroid cancer subtypes for validating the generalizability and diagnostic specificity of the proposed methods.

## 5 Conclusion

This study demonstrates that quantitative SHG microscopy can reliably distinguish normal from cancerous human thyroid tissue using label-free imaging of standard FFPE slides. Among the 35 collagen metrics evaluated, 19 demonstrated statistically significant discrimination between groups. Five additional metrics exhibited consistent directional trends, and six others showed effect sizes approaching |d|=0.8, revealing robust and biologically meaningful differences in collagen architecture despite the limited cohort size.

The novelty of this work lies in the systematic integration of geometric, intensity, textural, and frequency-domain SHG features, providing a multidimensional, interpretable collagen signature that conventional cytology and histology cannot capture. These results establish quantitative SHG as a promising foundation for an automated, real-time optical biopsy approach. Such a tool could reduce indeterminate FNAs, limit unnecessary repeat procedures and surgeries, and improve diagnostic confidence in thyroid cancer care.

Further validation in larger cohorts and across additional thyroid cancer subtypes will help advance this label-free quantitative imaging method toward clinical translation.

## Data Availability

The data supporting the findings of this study are available from the authors upon reasonable request. Given the absence of established standards for these measurements, public release of the data and code would not offer additional interpretive value for this work.
